# Data in support of optimized production of angiotensin-I converting enzyme inhibitory peptides derived from proteolytic hydrolysate of bitter melon seed proteins

**DOI:** 10.1016/j.dib.2015.09.038

**Published:** 2015-10-09

**Authors:** Anugerah Dany Priyanto, Robert J. Doerksen, Chi-I Chang, Wang-Chou Sung, Simon Bambang Widjanarko, Joni Kusnadi, Ya-Chi Lin, Ting-Chin Wang, Jue-Liang Hsu

**Affiliations:** a Department of Biological Science and Technology, National Pingtung University of Science and Technology, Pingtung 91201, Taiwan; b Department of Food Science, Faculty of Agricultural Technology, University of Brawijaya, Malang 65145, Indonesia; c Department of BioMolecular Sciences and Research Institute of Pharmaceutical Sciences, University of Mississippi, MS 38677, United States; d Vaccine Research and Development Center, National Health Research Institutes, Miaoli 35053, Taiwan; e Research Center for Tropic Agriculture, National Pingtung University of Science and Technology, Pingtung 91201, Taiwan; f Research Center for Austronesian Medicine and Agriculture, National Pingtung University of Science and Technology, Pingtung 91201, Taiwan

## Abstract

VY-7 has been demonstrated as a potent ACE inhibitory peptide in the previous study [1]. In this article, we provide accompanying data about the identification of bitter melon seed proteins (BMSPs), and quantitative analysis and optimized production of VY-7 in BMSPs hydrolysate.

## 1 **Specifications table**

**Table:** 

Subject area	Chemistry, Biology
More specific subject area	Angiotensin-I converting enzyme inhibitory peptides
Type of data	Tables of identified sequences and peptide yield enhancement; text description of the data; and figures of ACE inhibitory activities, LC–MS/MS and HPLC for BMSP hydrolysates
How data was acquired	Mass spectrometry, database search, ACE inhibitory assay, SDS-PAGE
Data format	Raw, filtered, and analyzed
Experimental factors	These are described in the text description of the data
Experimental features	These are described in the text description of the data
Data source location	Pingtung, Taiwan
Data accessibility	Data is supplied in this article

## 2 **Value of the data**

• Peptides identified from hydrolysate of bitter melon (*Momordica charantia*) seed proteins could be developed into food products.
• The approach we took to quantitative analysis of active peptides using LC–MS/MS under the mode of multiple reaction monitoring could be useful to others.
• Others interested in how to optimize peptide yield from natural sources could learn from our approach to tuning hydrolysis conditions, monitored by HPLC.

## 3 **Data**

ACE inhibitory activities of five BMSP hydrolysates (which were derived from trypsin, α-chymotrypsin, pepsin, alcalase, and thermolysin) were evaluated using *in vitro* ACE inhibitory assay. The identified peptides were analyzed using LC–MS/MS. The yields of VY-7 at various hydrolysis conditions were examined using LC–MS/MS (MRM mode) and HPLC.

## 4 **Experimental design, materials and methods**

### 4.1 Peptide identification using in-gel digestion, LC–MS/MS analysis and database matching

Bitter melon seed proteins (BMSPs) were separated using SDS-PAGE to give several distinct bands [1]. Five protein bands were in-gel digested by trypsin and the resulting peptides were analyzed using LC–MS/MS and Mascot database search. The data indicated that momordin A (protein source of VY-7) was located at the most intensive band positions with molecular weight around 28 kDa. The identified peptides derived from this protein are summarized in Table S1 in Supplementary information.

### 4.2 ACE inhibitory assay of BMSPs hydrolysates derived from different enzymes.

**Fig. 1:**
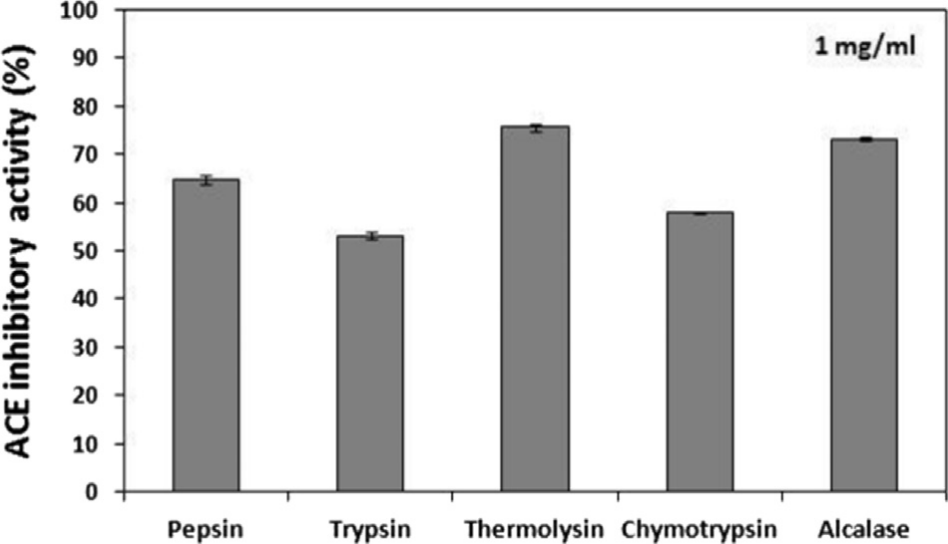
ACE inhibitory activities of the BMSP hydrolysates digested using different proteolytic enzymes. The hydrolysates derived from different enzymes were ultrafiltrated using 3 kDa MWCO ultrafiltration membranes and the resulting filtrates were lyophilized and subjected individually to ACE inhibitory assay.

BMSPs were digested using different proteolytic enzymes, trypsin (pH 8, 37 °C), α-chymotrypsin (pH 8, 37 °C), pepsin (pH 1.5, 37 °C), alcalase (pH 8, 50 °C), and thermolysin (pH 8, 60 °C). The hydrolysates derived from different enzymes were ultrafiltrated using 3 kDa MWCO ultrafiltration membranes and the resulting filtrates were lyophilized and subjected individually to ACE inhibitory assay according to previous report [2]. Data is shown in Fig. 1.

### 4.3 Quantitative analysis of VY-7 in BMSPs hydrolysate using LC–MS/MS.

**Fig. 2:**
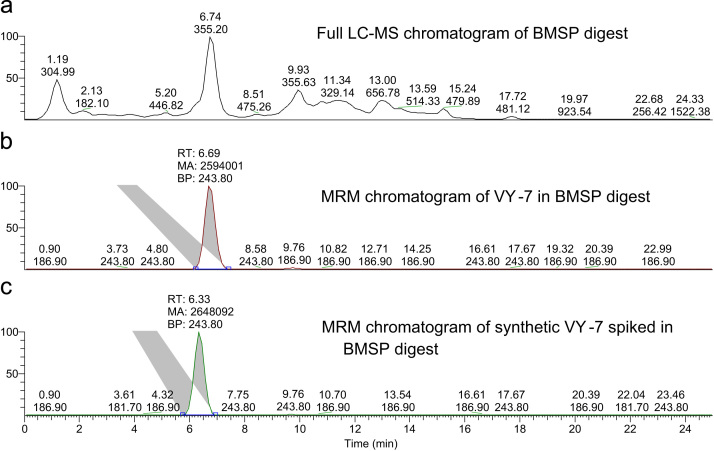
LC–MS/MS chromatograms. (A) Full chromatogram of the BMSP thermolysin digest; (B) MRM of VY-7 in the crude thermolysin digest of BMSPs; and (C) MRM chromatogram of synthetic VY-7 spiked in the BMSP thermolysin digest.

To determine the content of VY-7 in crude BMSPs hydrolysate, multiple reaction monitoring (MRM) was performed in LC–MS/MS analysis of BMSPs hydrolysate, similar to our previous report [3]. The MRM transition of VY-7 was set to be *m*/*z* 709.7>181.7 and 243.8 under a collision-induced dissociation (CID) energy of 40 V. Using the same LC–MS/MS condition, the calibration curve was established based on six standard peptide solutions at concentrations from 50 pg/µL to 25 ng/µL. The content of VY-7 in each BMSPs hydrolysate was determined according to its peak area in the MRM chromatogram. The full scan and VY-7 MRM chromatogram of BMSP hydrolysate are shown in Fig. 2. Moreover, the standard peptide was also spiked into crude BMSPs hydrolysate to confirm the quantification of the results based on the retention time and the spike yield (based on peak area), as shown in Fig. 2(C).

### 4.4 Optimization of target peptide formation by tuning hydrolysis conditions.

**Fig. 3:**
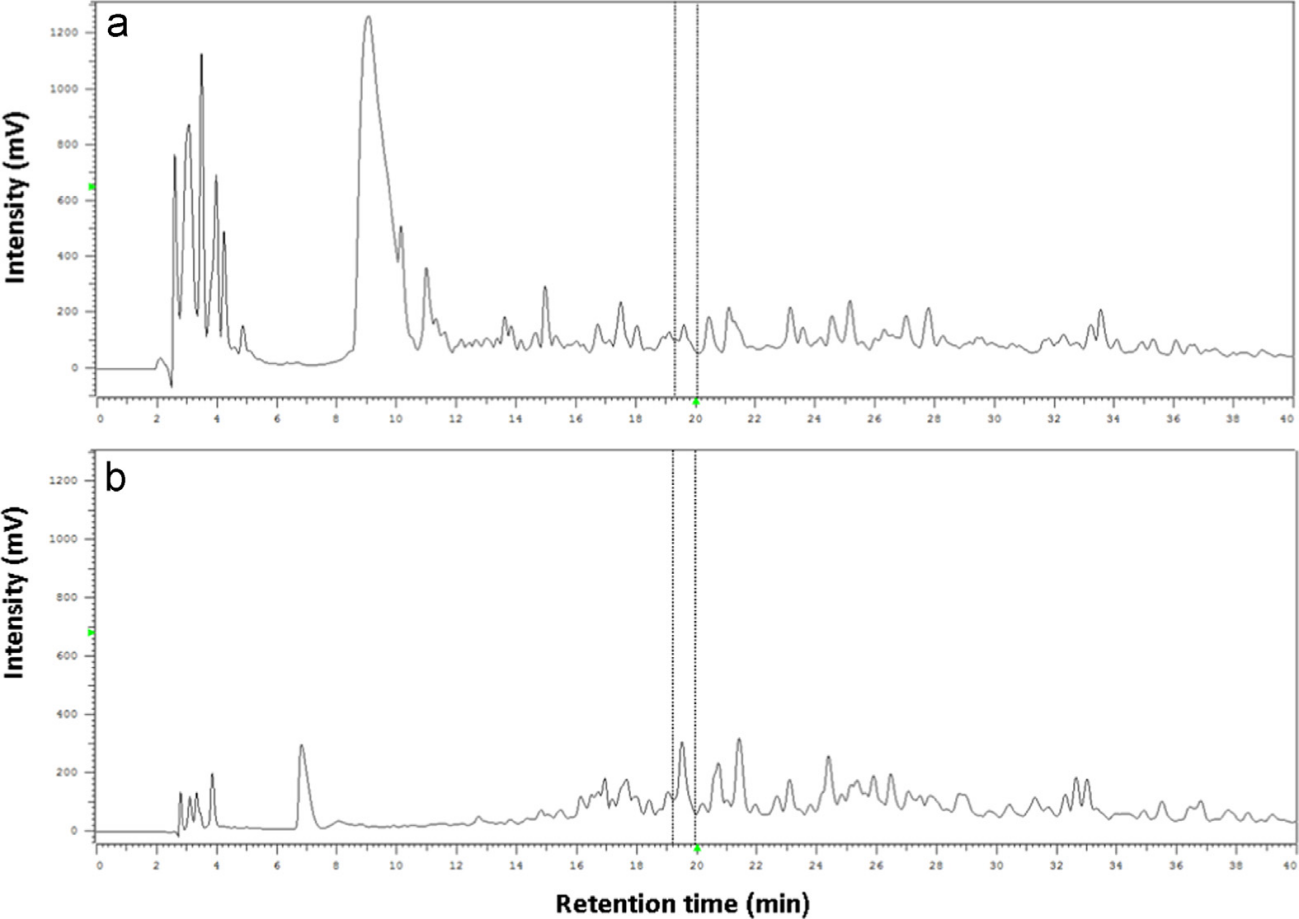
HPLC chromatograms of the BMSP thermolysin hydrolysate digested using condition (A) before; and (B) after optimization. The target peptide VY-7 is located between the two dotted lines.

To increase the yield of VY-7, the hydrolysis conditions, in terms of enzyme-to-protein ratios (w/w) (1/50, 1/100, 1/200, 1/400, and 1/800), hydrolysis times (1, 3, 6, 9, 12, and 15 h) and temperatures of incubation (40, 50, 60, and 70 °C), were optimized, using an approach similar to our previous report [4]. The optimum condition was defined as the condition which could produce the highest amount of target peptide. The yield of VY-7 in each hydrolysis condition was calculated based on MRM analysis, as mentioned in Section 4.3. The results are shown in Table 1. Using the optimum condition, an *E*/*S* ratio of 1:100 (w/w) was obtained with hydrolysis time of 12 h at 60 °C and 14.89±0.88 μg of VY-7 can be formed from 1 mg of BMSPs. The HPLC peptide patterns before and after hydrolysis optimization are shown in Fig. 3. The yield of target peptide was dramatically improved when the optimum conditions were used.

## Figures and Tables

**Table 1:** Effects of enzyme to substrate ratio (w/w), hydrolysis time (h), and temperature (°C) on peptide amount (μg/mg).

***E*/*S* ratio**	**Hydrolysis time (h)**	**Temperature (**°**C)**	**Peptide amount (μg/mg)**
1:50	12	60	10.20±0.84
1:100	14.89±0.88
1:200	10.65±0.85
1:400	4.21±0.44
1:800	2.78±0.05
1:100	1	2.05±0.25
3	3.96±0.79
6	7.77±0.48
9	10.34±0.68
15	2.48±0.26
12	40	4.42±0.23
50	8.05±0.27
70	1.49±0.05

## Footnotes

Appendix A Supplementary data associated with this article can be found in the online version at doi:10.1016/j.dib.2015.09.038.
