# Supplementary material for: Data in support of optimized production of angiotensin-I converting enzyme inhibitory peptides derived from proteolytic hydrolysate of bitter melon seed proteins
Source: Data Brief. 2015 Oct 9;5:403–7. doi: 10.1016/j.dib.2015.09.038 (PMC4773410; doi:10.1016/j.dib.2015.09.038)
Supplement: Supplementary file 1 — Supplementary material [file mmc1.doc]

*Supplementary Information*

**Table S1.** Summary of identified proteins and peptides from in-gel trypsin digestion of the band around 28 kDa using SDS-PAGE. The highlighted sequence is the peptide sequence of VY-7 from momordin A (alpha-momorcharin).

**Table S1.**

| Identified protein | Protein score | Identified peptide | Position start-end | Observed m/z | Charge number  z | Peptide mass (Calc) | Mascot score of peptide |
| --- | --- | --- | --- | --- | --- | --- | --- |
| Alpha-Momorcharin (Protein mass: 27.351 kDa) (GI:157829879) (Sequence coverage = 79.7%) | 859 | LSGADPR | 6-12 | 358.8 | 2 | 714.4 | 51 |
|  |  | VYNIPLLLPSVSGAGR | 31-46 | 829.1 | 2 | 1654.9 | 75 |
|  |  | NIPLLLPS**VSGAGR** | 33-46 | 697.9 | 2 | 1392.8 | 63 |
|  |  | **Y**LLMHLFNYDGK | 47-58 | 758.0 | 2 | 1512.7 | 71 |
|  |  | LFNYDGK | 52-58 | 429.0 | 2 | 855.4 | 42 |
|  |  | FFNEPAAELASQY | 82-94 | 744.0 | 2 | 1485.7 | 45 |
|  |  | FFNEPAAELASQYVFR | 82-97 | 945.1 | 2 | 1887.9 | 52 |
|  |  | KITLPY | 102-107 | 368.2 | 2 | 733.4 | 42 |
|  |  | KITLPYSGNYER | 102-113 | 481.6 | 3 | 1439.7 | 58 |
|  |  | ITLPYSGNYER | 103-113 | 657.1 | 2 | 1311.7 | 41 |
|  |  | LQIAAGK | 114-120 | 351.3 | 2 | 699.4 | 56 |
|  |  | LQIAAGKPR | 114-122 | 477.6 | 2 | 952.6 | 49 |
|  |  | EKIPIGLPALDSAISTL | 123-139 | 869.2 | 2 | 1737.0 | 54 |
|  |  | IPIGLPALDSAISTLLHY | 125-142 | 947.8 | 2 | 1893.0 | 48 |
|  |  | IPIGLPALDSAISTLLHYDST | 125-145 | 1099.4 | 2 | 2196.2 | 69 |
|  |  | IPIGLPALDSAISTLLHYDSTAAAGALL | 125-152 | 1383.6 | 2 | 2763.5 | 51 |
|  |  | ISTLLHYDSTAAAGALLVLIQTTAEAAR | 136-163 | 958.1 | 3 | 2869.6 | 51 |
|  |  | LHYDSTAAAGALLVLIQTTAEAAR | 140-163 | 1229.51 | 2 | 2455.3 | 81 |
|  |  | HYDSTAAAGALLVLIQTTAEAAR | 141-163 | 1172.81 | 2 | 2342.2 | 42 |
|  |  | AAAGALLVLIQTTAEAAR | 146-163 | 870.9 | 2 | 1738.9 | 66 |
|  |  | LVLIQTTAEAAR | 152-163 | 644.3 | 2 | 1284.7 | 65 |
|  |  | VLIQTTAEAAR | 153-163 | 587.3 | 2 | 1171.7 | 84 |
|  |  | YIEQQIQER | 166-174 | 604.2 | 2 | 1205.60 | 49 |
|  |  | AYRDEVPSLATISLENSWSGLSK | 175-197 | 1262.8 | 2 | 2522.3 | 87 |
|  |  | DEVPSLATISLENSWSGL | 178-195 | 960.0 | 2 | 1916.9 | 43 |
|  |  | QIQLAQGNNGIFR | 198-210 | 730.2 | 2 | 1457.8 | 40 |
|  |  | LAQGNNGIFR | 201-210 | 546.0 | 2 | 1088.6 | 83 |
|  |  | TPIVLVDNK | 211-219 | 500.3 | 2 | 997.6 | 71 |
|  |  | VQITNVTSK | 223-231 | 495.7 | 2 | 988.6 | 55 |
|  |  | VVTSNIQLLLNTR | 232-244 | 736.5 | 2 | 1469.9 | 101 |
|  |  | IQLLLNTR | 237-244 | 486.1 | 2 | 969.6 | 46 |
| Momordica Anti-HIV Protein 30 (Protein mass: 29.583 kDa) (GI:159162194) (Sequence coverage 69.2%) | 814 | DVNFDLSTATAK | 1-12 | 642.0 | 2 | 1280.6 | 66 |
|  |  | VYDIPLLYSTISDSR | 31-45 | 872.1 | 2 | 1740.9 | 115 |
|  |  | ESPPEAYNILFK | 85-96 | 705.3 | 2 | 1406.7 | 51 |
|  |  | KITLPY | 100-105 | 368.2 | 2 | 733.4 | 42 |
|  |  | KITLPYTGNYENLQTAAHK | 100-118 | 721.8 | 3 | 2161.1 | 68 |
|  |  | ITLPYTGNYENLQTAAH | 101-117 | 954.1 | 2 | 1904.9 | 120 |
|  |  | ITLPYTGNYENLQTAAHK | 101-118 | 1018.3 | 2 | 2033.0 | 32 |
|  |  | PYTGNYENLQTAAHK | 104-118 | 854.2 | 2 | 1705.8 | 71 |
|  |  | TGNYENLQTAAHK | 106-118 | 724.5 | 2 | 1445.7 | 63 |
|  |  | SAPSALLVLIQTTAEAAR | 144-161 | 906.6 | 2 | 1811.0 | 74 |
|  |  | APSALLVLIQTTAEAAR | 145-161 | 863.0 | 2 | 1723.9 | 41 |
|  |  | LVLIQTTAEAAR | 150-161 | 644.3 | 2 | 1284.7 | 65 |
|  |  | VLIQTTAEAAR | 151-161 | 587.3 | 2 | 1171.7 | 84 |
|  |  | YVATNFKPN | 172-180 | 527.6 | 2 | 1052.5 | 55 |
|  |  | YVATNFKPNLAIISLENQWSALSK | 172-195 | 1354.9 | 2 | 2706.4 | 51 |
|  |  | VATNFKPNLAIISLENQWSALSK | 173-195 | 849.3 | 3 | 2543.4 | 42 |
|  |  | FKPNLAIISLENQWSALSK | 177-195 | 720.9 | 3 | 2158.2 | 48 |
|  |  | KPNLAIISLENQWSALSK | 178-195 | 1007.2 | 2 | 2011.1 | 53 |
|  |  | PNLAIISLENQWSALSK | 179-195 | 943.1 | 2 | 1883.0 | 110 |
|  |  | LAIISLENQWSALSK | 181-195 | 837.4 | 2 | 1671.9 | 78 |
|  |  | QIFLAQNQGGK | 196-206 | 602.7 | 2 | 1202.6 | 67 |
|  |  | NPVDLIKPTGER | 209-220 | 670.2 | 2 | 1337.7 | 58 |
|  |  | FQVTNVDSDVVK | 221-232 | 676.5 | 2 | 1349.6 | 51 |
|  |  | SDVVKGNIKLLLNSR | 228-242 | 829.1 | 2 | 1654.9 | 50 |
| ribosomal inactivating protein [Momordica charantia] (Protein mass: 29.064 kDa) (GI:60459323) (Sequence coverage = 70%) | 587 | LSGADPR | 006-12 | 358.7 | 2 | 714.3 | 51 |
|  |  | VYNIPLLLPSVSGAGR | 31-46 | 829.1 | 2 | 1654.9 | 75 |
|  |  | NIPLLLPSVSGAGR | 33-46 | 697.9 | 2 | 1392.8 | 63 |
|  |  | FFNEPAAELASQY | 82-94 | 744.0 | 2 | 1485.6 | 45 |
|  |  | FFNEPAAELASQYVFR | 82-97 | 945.1 | 2 | 1887.9 | 52 |
|  |  | KITLPY | 102-107 | 368.2 | 2 | 733.4 | 42 |
|  |  | KITLPYSGNYER | 102-113 | 481.6 | 3 | 1439.7 | 58 |
|  |  | LQIAAGK | 114-120 | 351.3 | 2 | 699.4 | 56 |
|  |  | LQIAAGKPR | 114-122 | 477.6 | 2 | 952.5 | 49 |
|  |  | EKIPIGLPALDSAISTL | 123-139 | 869.9 | 2 | 1736.9 | 54 |
|  |  | IPIGLPALDSAISTLLHY | 125-142 | 947.8 | 2 | 1893.1 | 48 |
|  |  | IPIGLPALDSAISTLLHYDST | 125-145 | 1099.4 | 2 | 2196.2 | 69 |
|  |  | IPIGLPALDSAISTLLHYDSTAAAGALL | 125-152 | 1383.6 | 2 | 2763.5 | 51 |
|  |  | YIEQQIQER | 166-174 | 604.2 | 2 | 1205.6 | 49 |
|  |  | AYRDEVPSLATISLENSWSGLSK | 175-197 | 1262.7 | 2 | 2522.2 | 87 |
|  |  | DEVPSLATISLENSWSGL | 178-195 | 960.0 | 2 | 1916.9 | 43 |
|  |  | QIQLAQGNNGIFR | 198-210 | 730.2 | 2 | 1457.8 | 40 |
|  |  | LAQGNNGIFR | 201-210 | 546.0 | 2 | 1088.6 | 83 |
|  |  | TPIVLVDNK | 211-219 | 500.3 | 2 | 997.5 | 71 |
|  |  | VQITNVTSK | 223-231 | 495.6 | 2 | 988.5 | 55 |
|  |  | VVTSNIQLLLNTR | 232-244 | 736.5 | 2 | 1469.8 | 101 |
|  |  | IQLLLNTR | 237-244 | 486.1 | 2 | 969.6 | 46 |
| Momordica charantia, Linn Cucurbitae Trypsin inhibitor-A (Protein mass: 24.143 kDa) (GI:494360) (Sequence coverage = 25.1%) | 97 | LGEHNIDVLEGNEQFINAAK | 50-69 | 738.3 | 3 | 2210.1 | 64 |
|  |  | TLDNDIMLIK | 80-89 | 596.3 | 2 | 1190.6 | 56 |
| galactose-binding type-2 ribosome-inactivating protein [Momordica charantia] (Protein mass: 61.639 kDa) (GI:219567000) (Sequence coverage = 2.9%) | 65 | IGNYVEPIVTTIIGLR | 416-431 | 880.0 | 2 | 1757.0 | 65 |
| RNA dependent RNA polymerase [Melon yellow spot virus] (Protein mass: 333.122 kDa) (GI:198446408) (Sequence coverage = 1%) | 46 | IAKGLMVADR | 743-752 | 546.0 | 2 | 1088.6 | 46 |

*Highlighted data represent the peptide sequence of VY-7 from the bitter melon protein
